# Anemia as an independent risk factor for sarcopenia in older adults: a cohort study based on CHARLS and ELSA

**DOI:** 10.1038/s41598-026-54626-6

**Published:** 2026-05-27

**Authors:** Zengqiang Liu, Weidong Jiang, Zhen Liu, Aiqiong Qin, Wen Liu, Xiaodi Sun, Fanfan Xu

**Affiliations:** 1 Department of Gerontology, The Second Qilu Hospital of Shandong University, Jinan, 250033 Shandong People’s Republic of China; 2 Department of Emergency, The Second Qilu Hospital of Shandong University, Shandong University, Jinan, 250033 Shandong People’s Republic of China

**Keywords:** Anemia, Sarcopenia, Cohort studies, Older adults, Diseases, Health care, Medical research, Risk factors

## Abstract

This study examined the prospective association between anemia and incident sarcopenia using two large-scale aging cohorts: the China Health and Retirement Longitudinal Study (CHARLS) and the English Longitudinal Study of Ageing (ELSA). This prospective, population-based cohort study included participants aged ≥ 45 years without sarcopenia at baseline from CHARLS (Wave 1, 2011) and ELSA (Wave 4, 2008–2009). Hazard ratios (HRs) were estimated using multivariable Cox proportional hazards models with sequential adjustment for demographic, socioeconomic, lifestyle, and health-related confounders. Subgroup analyses and sensitivity analyses were conducted to test robustness. After full adjustment, baseline anemia was significantly associated with an increased risk of incident sarcopenia in both CHARLS (*n* = 1,407; HR = 1.73, 95% CI: 1.07–2.79) and ELSA (*n* = 2,921; HR = 2.62, 95% CI: 1.50–4.56). In ELSA, a stronger association was observed among females (HR = 4.72, 95% CI: 2.25–9.93), with a marginal sex interaction (P for interaction = 0.054). No significant sex interaction was detected in CHARLS.Sensitivity analyses using sequential adjustment models confirmed consistent results (CHARLS: HR = 1.53, 95% CI: 1.17–2.01; ELSA: HR = 2.59, 95% CI: 1.30–5.18). This bicohort study suggests that baseline anemia is associated with a higher risk of developing sarcopenia in older adults from two distinct populations (Chinese and British). The strength of this association and the susceptible subgroups differed between cohorts. Further studies are needed to determine whether correcting anemia can reduce sarcopenia incidence and whether risk stratification based on anemia improves prevention strategies.

**Supplementary Information:**

The online version contains supplementary material available at 10.1038/s41598-026-54626-6.

## Introduction

Sarcopenia, defined as the progressive and generalized loss of skeletal muscle mass and strength, stands as a leading contributor to frailty, disability, and premature mortality in aging populations worldwide^1^. Its rising prevalence constitutes a significant global public health challenge^2^. Concurrently, anemia is a highly prevalent comorbidity in older adults, often attributable to nutritional deficiencies, chronic inflammation, or renal dysfunction^3^. Beyond its well-established association with fatigue, anemia serves as an independent predictor of diminished physical performance and increased mortality risk in this population^4^.

Given the high prevalence of both conditions in geriatric settings and the potential for anemia to be a modifiable risk factor, clarifying this relationship is critical for developing targeted prevention strategies in older adults. A biologically plausible mechanistic link underpins the association between these two conditions. Hemoglobin plays a critical role in oxygen delivery to muscle tissue. Insufficient oxygen supply disrupts mitochondrial respiration and protein synthesis, thereby accelerating muscle atrophy^5^. Additionally, chronic inflammation—a shared pathophysiological mechanism—simultaneously disrupts erythropoiesis and induces muscle catabolism via pro-inflammatory cytokines^6^. While cross-sectional studies have consistently demonstrated associations between lower hemoglobin levels and reduced muscle mass and strength^7,8^, the temporal relationship remains unresolved. A pivotal unanswered question thus remains: Whether anemia constitutes an independent risk factor for incident sarcopenia? This uncertainty stems from the inability of cross-sectional designs to exclude reverse causality, wherein deteriorating muscle health may secondarily suppress hemoglobin levels^9^.

A recent systematic review and meta-analysis confirmed the association between lower hemoglobin levels and sarcopenia but highlighted the predominance of cross-sectional designs and the pressing need for prospective evidence to establish causality^9^. Furthermore, dysregulated iron metabolism, a key contributor to anemia, has been directly linked to impaired muscle mass and function in clinical studies^10^, reinforcing the biological plausibility of the anemia-sarcopenia link. However, prospective evidence specifically linking anemia(a clinical manifestation) to incident sarcopeniaremains limited, and comparisons across diverse populations are scarce.

To address this gap, our study utilizes two large-scale longitudinal cohorts from Eastern and Western populations—the China Health and Retirement Longitudinal Study (CHARLS)^11^ and the English Longitudinal Study of Ageing (ELSA)^12^. By employing harmonized analytical approaches in parallel within the Chinese CHARLS and British ELSA populations, this bicohort study is uniquely positioned not only to prospectively examine this relationship but also to directly compare its strength and modifiers across two distinct cross-population and socioeconomic contexts, offering insights beyond single-population or retrospective studies.

## Methods

### Study design and data sources

This prospective cohort study utilized data from two independent, nationally representative longitudinal surveys: the China Health and Retirement Longitudinal Study(CHARLS) and the English Longitudinal Study of Ageing(ELSA). We selected these two cohorts because both are nationally representative studies of aging with repeated follow-ups, and they collect detailed data on physical function, venous blood biomarkers, and a wide range of covariates. This allows for the harmonized definition of baseline anemia, prospective ascertainment of incident sarcopenia, and adjustment for key confounders, enabling parallel analyses in two distinct older populations.

For CHARLS, the 2011 wave (Wave 1) served as the baseline. For ELSA, the 2008–2009 wave (Wave 4) was used as the baseline, as it was the first wave with comprehensive hemoglobin measurements required for anemia assessment. To ensure comparability, participants aged ≥ 45 years from both cohorts were eligible for inclusion. Participants were included if they: (1) had complete data for defining baseline anemia (hemoglobin), sarcopenia, and key covariates; and (2) were free of sarcopenia at baseline. Participants with prevalent sarcopenia at baseline or missing follow-up data were excluded.

**Fig. 1:**
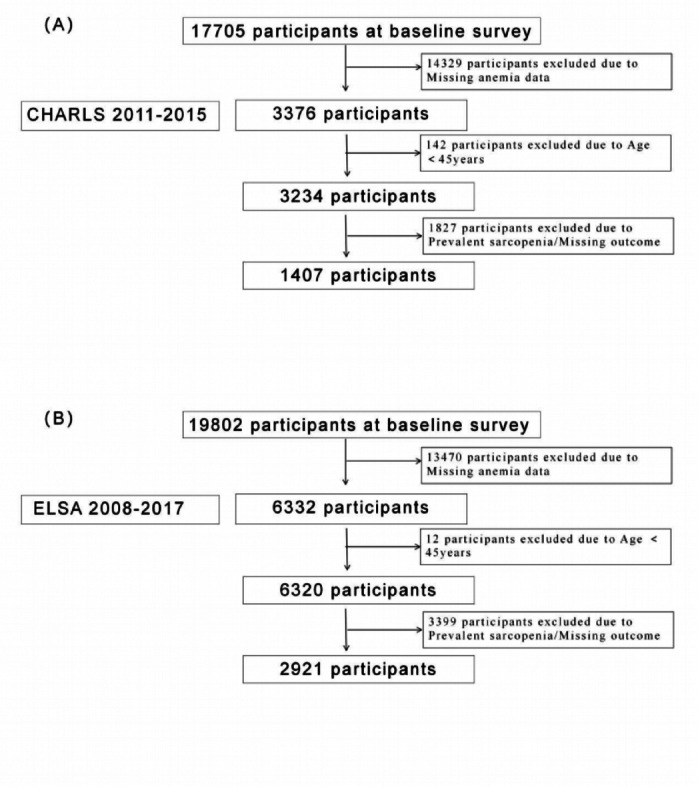
Study flow diagram of participant selection in CHARLS and ELSA cohorts.

A detailed flowchart of participant selection is presented in Fig. 1, showing the exclusion process and final sample sizes for both CHARLS and ELSA cohorts.

The exposure (anemia) and all covariates were assessed only at baseline. Participants were then followed for the assessment of incident sarcopenia at subsequent waves: in CHARLS at Waves 2 (2013) and 3 (2015); in ELSA at Waves 6 (2012–2013) and 8 (2016–2017).

Definition of Time-to-Event for Survival Analysis: The primary outcome was time to incident sarcopenia. For participants who developed sarcopenia, the event time was defined as the time (in years) from the baseline assessment to the first follow-up wave at which they met the diagnostic criteria for sarcopenia. Given the interval between survey waves, the exact onset time was interval-censored; this approach effectively assigns the event time at the midpoint between the last non-sarcopenic and first sarcopenic assessment, which is a standard method for handling such data. For those who did not develop sarcopenia during the study period, their follow-up time was censored at the date of their last available sarcopenia assessment (Wave 3 for CHARLS, Wave 8 for ELSA). The timevariable in the subsequent Cox models corresponds to this defined survival time.

All participants provided written informed consent.

### Variable definitions

#### Exposure variable: anemia

Anemia was defined according to World Health Organization (WHO) criteria: hemoglobin concentration < 12 g/dL for non-pregnant women and < 13 g/dL for men^14^. Hemoglobin levels were measured from venous blood samples using standardized protocols (CHARLS: Mindray BC-2800 analyzer; ELSA: Beckman Coulter LH 750 analyzer)^12,13^. All anthropometric and laboratory measurements were conducted by trained personnel with inter-rater reliability > 0.85, as per geriatric assessment standards.

#### Outcome variable: sarcopenia

Sarcopenia was diagnosed using cohort-specific criteria to account for regional and methodological differences:

##### CHARLS:

Applied the Asian Working Group for Sarcopenia 2019 (AWGS 2019) criteria^2^. Low muscle strength was defined as handgrip strength < 28 kg for men and < 18 kg for women (Yuejian WL-1000 dynamometer). Low muscle mass was defined as appendicular skeletal muscle mass index (ASM/height²) < 6.90 kg/m² for men and < 5.16 kg/m² for women, estimated via a validated anthropometric equation. Poor physical performance was defined as gait speed < 1.0 m/s or five-time chair stand test ≥ 12 s. Sarcopenia was defined as having low muscle mass, in addition to either low muscle strength or poor physical performance.

##### ELSA:

Applied the European Working Group on Sarcopenia in Older People 2 (EWGSOP2) criteria^1^. Low muscle strength was defined as handgrip strength < 27 kg for men and < 16 kg for women (Smedley dynamometer). Low muscle mass was defined as ASM/height² <7.0 kg/m² for men and < 5.5 kg/m² for women, measured via bioelectrical impedance analysis (BIA; Tanita BC-418MA). Poor physical performance was defined as gait speed < 0.8 m/s. These cohort-specific criteria align with recommendations from the FNIH sarcopenia project^14^, ensuring alignment with international standards^15^.

Incident sarcopenia was defined as the first occurrence during follow-up among participants without sarcopenia at baseline. All measurements followed standardized protocols with inter-rater reliability > 0.85.

#### Covariates

A set of potential confounders assessed at baseline were included. To enhance comparability, variable definitions were harmonized between cohorts where feasible. The specific definitions are as follows:

**Demographic and Socioeconomic Factors**

Age was analyzed as a continuous variable (years).

Sex was categorized as male or female.

Marital status was harmonized as binary: Married versus Single across both cohorts.

Education was harmonized into a binary classification reflecting educational attainment: “Up to secondary” versus “Above secondary”.

Household economic status was used as a proxy for socioeconomic status. In CHARLS, this was defined as annual household economic status (Chinese Yuan, CNY). In ELSA, it was defined as annual household economic status (British Pounds, GBP). Both were treated as continuous variables in the models.

**Lifestyle and Anthropometric Factors**

Smoking status was fully harmonized as “Ever smoker” (encompassing both current and former smokers) versus “Never smoker”.

Drinking status was fully harmonized as “Ever drinker” versus “Never drinker”.

Body mass index (BMI) was calculated as weight in kilograms divided by height in meters squared (kg/m²) and analyzed as a continuous variable. For subgroup analysis, obesity was defined using cohort-specific and population-appropriate cut-offs: BMI ≥ 24 kg/m² (using Asian criteria) in CHARLS and BMI ≥ 25 kg/m² (using standard WHO criteria) in ELSA.

**Health Status Factors**

Number of chronic conditions was based on a count of self-reported, physician-diagnosed diseases (11 conditions in CHARLS, e.g., asthma, cancer, lung disease, heart problem, arthritis; 13 conditions in ELSA, e.g., cancer, angina, heart attack, stroke, arthritis). Both cohorts were dichotomized as No (none) or Yes (at least one) in the models.

Activities of daily living (ADL) disability score was assessed as the number of tasks with which participants reported any difficulty. In CHARLS, this was assessed via a 6-item scale (including dressing, bathing, eating, getting in/out of bed, using the toilet, and controlling urination and defecation), with the total score ranging from 0 to 6^11^. In ELSA, this was assessed via a harmonized 5-item ADL scale (including dressing, walking across a room, bathing, eating, and getting in or out of bed), with the total score ranging from 0 to 5^16^. A higher score indicates greater functional limitation.

Baseline characteristics including age, sex, marital status, educational attainment, annual household economic status, smoking and drinking status, body mass index (BMI), number of chronic conditions, and activities of daily living (ADL) disability score were collected and are presented in Tables 1 and 2. The follow-up duration for each participant was calculated from the baseline visit to the occurrence of sarcopenia, loss to follow-up, or the end of the study period, and is reported as mean ± standard deviation.

**Table 1:** Baseline characteristics of the study participants in the CHARLS cohort.

Characteristic	Level	Overall (*N* = 1407)	Non-anemia (*N* = 1169)	Anemia (*N* = 238)	*p*-value
Age, years	Mean ± SD	57.98 ± 8.30	57.60 ± 7.99	59.84 ± 9.45	< 0.001
Sex, n (%)	Female	682 (48.5)	555 (47.5)	127 (53.4)	0.113
	Male	725 (51.5)	614 (52.5)	111 (46.6)	
Residence, n (%)	Rural	1011 (71.9)	849 (72.6)	162 (68.1)	0.178
	Urban	396 (28.1)	320 (27.4)	76 (31.9)	
Marital status, n (%)	Married	1249 (88.8)	1033 (88.4)	216 (90.8)	0.266
	Single	158 (11.2)	136 (11.6)	22 (9.2)	
Education, n (%)	Up to secondary	1072 (76.2)	895 (76.6)	177 (74.4)	0.522
	Above secondary	335 (23.8)	274 (23.4)	61 (25.6)	
Annual household economic status, CNY	Mean ± SD	6700.06 ± 6551.48	6888.51 ± 6608.60	5755.00 ± 6186.51	0.024
Smoking status, n (%)	Never smoker	829 (59.2)	682 (58.5)	147 (62.6)	0.285
	Ever smoker	571 (40.8)	483 (41.5)	88 (37.4)	
Drinking status, n (%)	Ever drinker	403 (30.1)	331 (29.8)	72 (31.7)	0.619
	Never drinker	935 (69.9)	780 (70.2)	155 (68.3)	
Body mass index, kg/m²	Mean ± SD	24.49 ± 13.38	24.69 ± 14.60	23.53 ± 2.93	0.239
Number of chronic conditions, n (%)	No	502 (35.7)	415 (35.5)	87 (36.6)	0.899
	Yes	905 (64.3)	754 (64.5)	151 (63.4)	
ADL disability score	Mean ± SD	0.29 ± 0.81	0.25 ± 0.74	0.46 ± 1.06	< 0.001

**Table 2:** Baseline characteristics of the study participants in the ELSA cohort.

Characteristic	Level	Overall (*N* = 2921)	Non-anemia (*N* = 2822)	Anemia (*N* = 99)	*p*-value
Age, years	Mean ± SD	63.42 ± 7.38	63.35 ± 7.33	65.61 ± 8.41	0.003
Sex, n (%)	Female	1590 (54.4)	1539 (54.5)	51 (51.5)	0.624
	Male	1331 (45.6)	1283 (45.5)	48 (48.5)	
Marital status, n (%)	Married	2104 (74.6)	2039 (74.8)	65 (67.7)	0.146
	Single	717 (25.4)	686 (25.2)	31 (32.3)	
Education, n (%)	Up to secondary	765 (28.3)	739 (28.3)	26 (28.3)	1
	Above secondary	1941 (71.7)	1875 (71.7)	66 (71.7)	
Annual household economic status, GBP	Mean ± SD	4728.15 ± 12826.26	4823.67 ± 13011.31	2035.93 ± 4747.08	0.034
Smoking status, n (%)	Never smoker	1238 (42.4)	1194 (42.4)	44 (44.4)	0.757
	Ever smoker	1680 (57.6)	1625 (57.6)	55 (55.6)	
Drinking status, n (%)	Ever drinker	2529 (93.0)	2447 (93.1)	82 (91.1)	0.601
	Never drinker	189 (7.0)	181 (6.9)	8 (8.9)	
Body mass index, kg/m²	Mean ± SD	27.87 ± 4.81	27.85 ± 4.75	28.39 ± 6.37	0.274
Number of chronic conditions, n (%)	No	929 (31.8)	904 (32.0)	25 (25.3)	0.189
	Yes	1992 (68.2)	1918 (68.0)	74 (74.7)	
ADL disability score	Mean ± SD	0.15 ± 0.51	0.14 ± 0.50	0.31 ± 0.80	0.001

### Statistical analysis

All statistical analyses were performed separately in the CHARLS and ELSA cohorts. Baseline characteristics were compared between participants with and without anemia. Student’s t-test was applied for continuous variables, and the chi-square test for categorical variables. Continuous variables were described as mean ± standard deviation (SD), while categorical variables were presented as number and percentage.

All confounders included in the regression models were uniformly defined in Sect. 2.2.3. Multivariable Cox proportional hazards regression models were constructed to calculate hazard ratios (HRs) and 95% confidence intervals (CIs) for the association between anemia and sarcopenia risk. Three hierarchical models were established for each cohort.In both cohorts, Model 1 was the unadjusted crude model. Model 2 was adjusted for demographic and socioeconomic factors: sex, marital status, education status, and annual household economic status. Model 3 was further adjusted for number of chronic conditions, body mass index (BMI), smoking status, drinking status, and ADL disability score.

The proportional hazards assumption was confirmed using Schoenfeld residuals, and no obvious violation was observed (all *P* > 0.05). Based on the fully adjusted Model 3, subgroup analyses were stratified by sex, smoking status, drinking status and obesity. Multiplicative interaction terms were introduced into the Cox model to test the interaction effect between anemia and each stratifying factor.

Multiple imputation by chained equations (MICE) was adopted to address missing covariate data, and sensitivity analysis was conducted by repeating the main Cox regression in imputed datasets to verify the robustness of primary results.

All statistical analyses were conducted using R software (version 4.5.0). A two-sided *P* < 0.05 was considered statistically significant.

## Results

Over a mean (SD) follow-up of 4.2 (1.1) years in CHARLS and 8.5 (2.3) years in ELSA, 112 and 89 incident sarcopenia cases were documented, respectively.

### Baseline characteristics of the study populations

Baseline characteristics stratified by anemia status are presented in Table 1 (CHARLS, *n* = 1,407) and Table 2 (ELSA, *n* = 2,921). In both cohorts, participants with anemia were significantly older and had higher ADL disability scores compared with those without anemia (all *P* < 0.01). Anemic participants also had lower annual household economic status (CHARLS: 5,755 vs. 6,889 CNY, *P* = 0.024; ELSA: 2,036 vs. 4,824 GBP, *P* = 0.034). Other demographic, lifestyle, and health-related characteristics were generally balanced between groups (all *P* > 0.05).

### Association between anemia and incident sarcopenia

Multivariable Cox proportional hazards regression models showed that baseline anemia was significantly associated with an increased risk of incident sarcopenia (Table 3). In the CHARLS cohort, the fully adjusted HR was 1.73 (95% CI: 1.07–2.79, *P* = 0.025). In the ELSA cohort, the association was stronger, with a fully adjusted HR of 2.62 (95% CI: 1.50–4.56, *P* < 0.001). The magnitude and direction of association were consistent across sequential adjustment models in both cohorts.

**Table 3:** Association between baseline anemia and incident sarcopenia: Results from Cox proportional hazards models.

Cohort (*n*)	Model	HR (95% CI)	*p*-value
CHARLS (1,407)	Model 1	1.43 (0.92, 2.22)	0.11
	Model 2	1.61 (1.01, 2.58)	0.044
	Model 3	1.73 (1.07, 2.79)	0.025
ELSA (2,921)	Model 1	2.95 (1.79, 4.87)	< 0.001
	Model 2	3.15 (1.90, 5.21)	< 0.001
	Model 3	2.62 (1.50, 4.56)	< 0.001

### Subgroup analyses

Subgroup analyses are presented in Supplementary Tables S1, S2 and Supplementary Figures S7, S8. Pronounced sex-related heterogeneity was observed between the two cohorts. In the CHARLS cohort, the association between baseline anemia and incident sarcopenia was significant only in men (HR = 2.08, 95% CI: 1.06–4.05, *P* = 0.032) but not in women (HR = 1.32, 95% CI: 0.68–2.58, *P* = 0.411). In the ELSA cohort, a strong significant association was observed only in women (HR = 4.72, 95% CI: 2.25–9.93, *P* < 0.001), whereas no significant association was found in men (HR = 0.98, 95% CI: 0.24–4.02, *P* = 0.979). Moreover, in ELSA, the association was significant only among participants with BMI ≥ 25 kg/m² (HR = 3.03, 95% CI: 1.58–5.79, *P* = 0.001), while the estimate for the BMI < 25 kg/m² subgroup was not estimable (NE) due to the limited number of anemic cases and outcome events. No significant effect modification by smoking status or drinking status was identified in either cohort (all P for interaction > 0.05).

### Sensitivity analyses

Sensitivity analyses using sequential adjustment models confirmed the robustness of the main findings. In CHARLS, the fully adjusted model yielded an HR of 1.53 (95% CI: 1.17–2.01, *P* = 0.002; Supplementary Table S3, Figure S5). In ELSA, the corresponding HR was 2.59 (95% CI: 1.30–5.18, *P* = 0.007; Supplementary Table S4, Figure S6).

### Kaplan–meier analysis of sarcopenia incidence

**Fig. 2:**
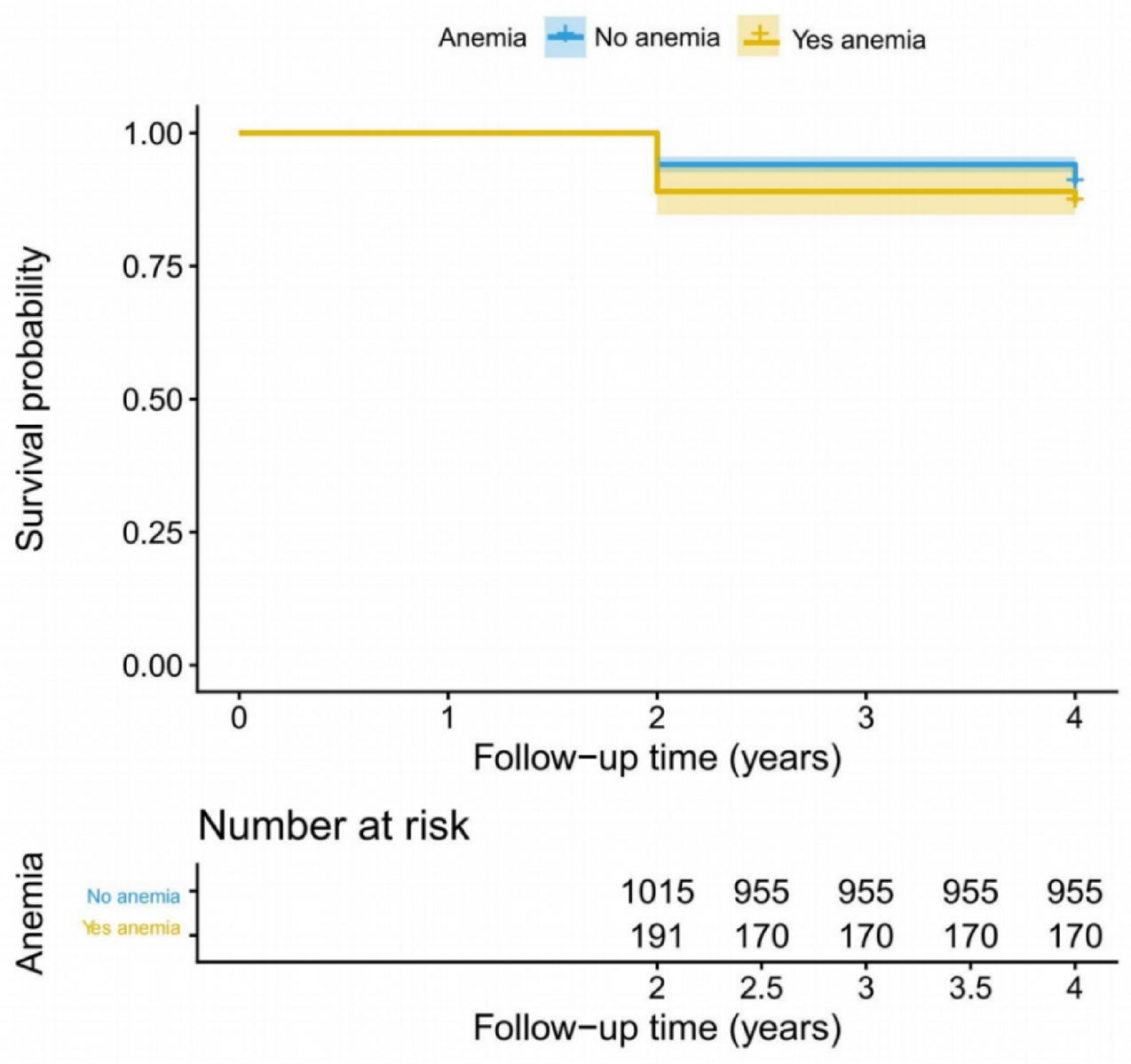
Kaplan-Meier curves for sarcopenia-free survival by baseline anemia status in the CHARLS cohort. Anemia was defined according to WHO criteria at baseline (2011). The primary outcome was incident sarcopenia during 4 years of follow-up. Blue line: participants without anemia; yellow line: participants with anemia. The number of participants at risk at each time point is shown below the curve.

**Fig. 3:**
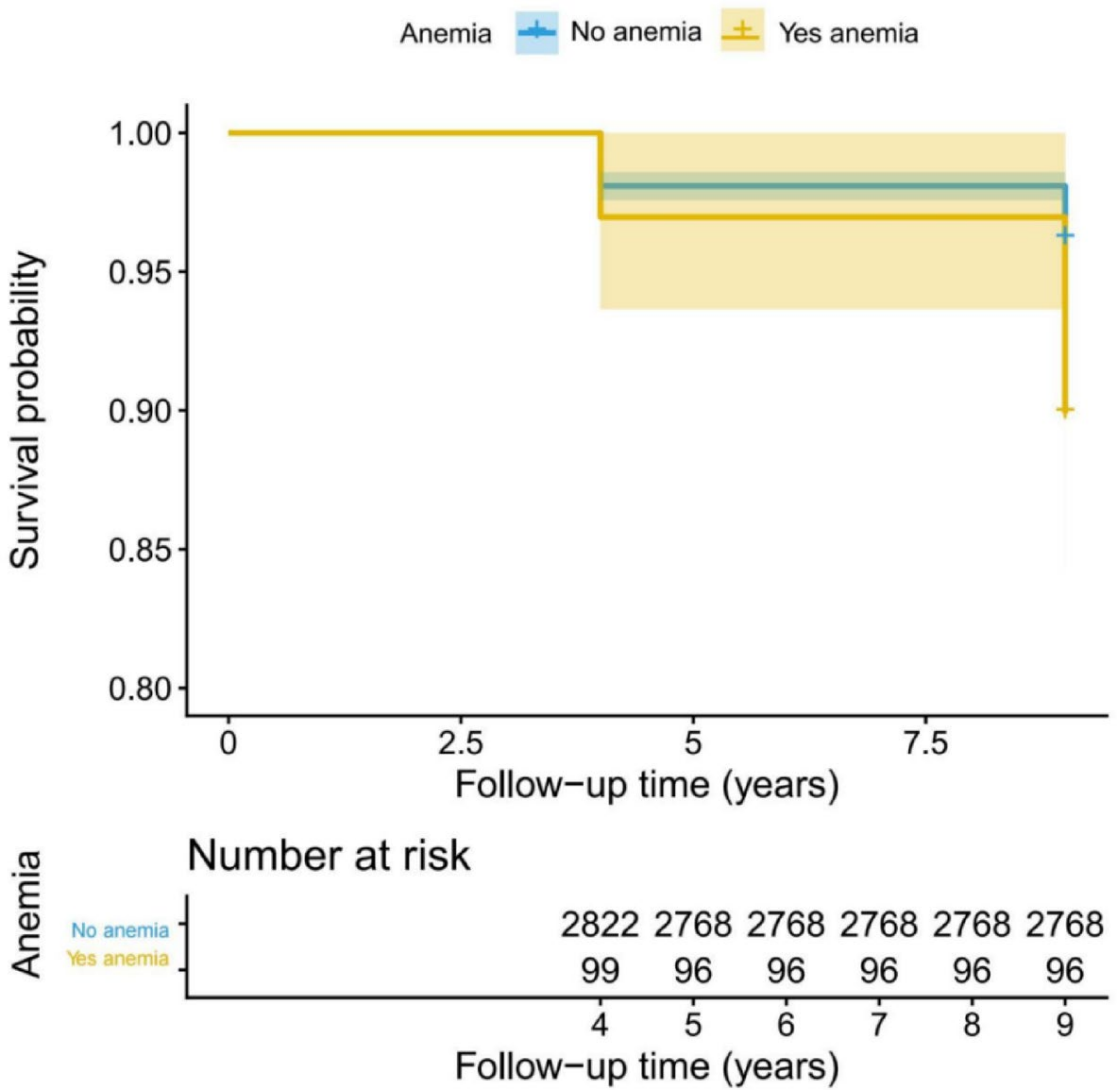
Kaplan-Meier curves for sarcopenia-free survival by baseline anemia status in the ELSA cohort. Anemia was defined according to WHO criteria at baseline (2008-2009). The primary outcome was incident sarcopenia during up to 9 years of follow-up. Blue line: participants without anemia; yellow line: participants with anemia. The number of participants at risk at each time point is shown below the curve.

Kaplan–Meier curves further illustrated the association between baseline anemia and incident sarcopenia (Figs. 2 and 3). In CHARLS (Fig. 2), the sarcopenia-free survival rate was consistently lower in the anemic group, with a significant difference by log-rank test (*P* < 0.001). In ELSA (Fig. 3), the curves separated early and remained divergent throughout follow-up, and the between-group difference was also significant (log-rank *P* < 0.001).

## Discussion

This prospective bicohort study demonstrates that baseline anemia is associated with an increased risk of incident sarcopenia among middle‑aged and older adults in both the Chinese CHARLS and British ELSA populations. After full adjustment (Model 3) for confounders, anemia was significantly associated with higher sarcopenia risk, with a stronger point estimate observed in ELSA (HR = 2.62) than in CHARLS (HR = 1.73). Subgroup analyses suggested potential sex‑related differences: in CHARLS, the association was significant in men but not women; in ELSA, the association was significant in women but not men. These observations indicate possible variability in the anemia–sarcopenia association across populations and sexes, although formal statistical tests of between‑cohort heterogeneity were not performed.

These findings align with previous cross‑sectional and longitudinal evidence linking lower hemoglobin levels to reduced muscle mass, strength, and physical performance in older adults^7,8,17^. A biologically plausible mechanism supports the observed association. Anemia reduces oxygen delivery to skeletal muscle, which may impair mitochondrial respiration and protein synthesis, thereby promoting muscle atrophy^5,27^. In addition, chronic inflammation, a common contributor to both anemia and sarcopenia, may simultaneously suppress erythropoiesis and enhance muscle catabolism through pro‑inflammatory cytokines^6,20,29^. Dysregulated iron metabolism, which is frequently involved in anemia, has also been associated with lower muscle mass and function^10,18^. Together, these pathways provide a biologically consistent basis for the observed prospective association.

Some between‑cohort differences in anemia prevalence and effect sizes were noted. The prevalence of anemia was higher in CHARLS (16.9%) than in ELSA (3.4%). Such differences are consistent with broader epidemiologic patterns reported in older Asian versus Western populations^24–26^. Variation in anemia etiology—including nutritional deficiencies, chronic inflammation, and renal dysfunction—may contribute to differences in effect magnitude across settings^3,18–23^. However, without formal statistical comparison of hazard ratios between cohorts, firm conclusions regarding cross-population differences in the strength of the anemia–sarcopenia association cannot be drawn.

Similarly, sex‑stratified analyses revealed divergent patterns across cohorts. In CHARLS, the association was significant among men; in ELSA, the association was significant among women. These subgroup findings suggest potential effect modification by sex, but the results were inconsistent between cohorts and interaction terms were of borderline or non‑significant magnitude. Accordingly, these observations should be considered exploratory and require replication in larger studies with sufficient statistical power for sex‑specific analyses^28^.

This study has several strengths. It uses a prospective design in two large, nationally representative aging cohorts, which minimizes recall bias and supports temporal inference. Harmonized definitions of anemia and consistent adjustment for major confounders enhance internal validity. Sensitivity analyses using sequential adjustment models confirmed consistent and stable effect estimates, further supporting the robustness of the main findings.

Several limitations should be acknowledged. First, anemia was defined only at baseline, and changes in hemoglobin status during follow‑up were not accounted for; thus, potential effects of anemia persistence or resolution could not be evaluated^17^. Second, the time to sarcopenia onset was treated as interval‑censored and assigned at the midpoint between waves, which may introduce some imprecision in event‑time estimation. Third, while multiple imputation was used to address missing data, the detailed proportion, pattern, and sensitivity of missing‑data handling were not fully reported. Fourth, no formal statistical test for between‑cohort heterogeneity was conducted; therefore, direct quantitative comparisons of effect sizes across CHARLS and ELSA are exploratory and should be interpreted with caution. Fifth, sarcopenia was defined using AWGS 2019 criteria in CHARLS and EWGSOP2 criteria in ELSA, with muscle mass estimated by anthropometric equations in CHARLS and measured by BIA in ELSA. These methodological differences may influence absolute event rates and comparability of effect estimates. Finally, subgroup analyses were limited by small numbers in some strata, which may reduce precision and stability of effect estimates^32^.

In conclusion, this prospective bicohort study shows that baseline anemia is associated with a higher risk of developing sarcopenia in middle‑aged and older adults in China and England. These results support anemia as a potential indicator of elevated sarcopenia risk in older populations. Further research is needed to confirm these findings in other populations, evaluate whether longitudinal changes in anemia influence sarcopenia risk, explore underlying biological mechanisms, and determine whether correcting anemia may help prevent sarcopenia in at‑risk individuals^17,30,31,33^.

## Supplementary Information

Below is the link to the electronic supplementary material.

Supplementary Material 1

Supplementary Material 2

Supplementary Material 3

Supplementary Material 4

Supplementary Material 5

Supplementary Material 6

Supplementary Material 7

Supplementary Material 8

## Data Availability

The datasets analyzed during the current study are available from the CHARLS and ELSA repositories.CHARLS data are publicly available at http://charls.pku.edu.cn/.ELSA data are available from the UK Data Service (https://ukdataservice.ac.uk/). Access to ELSA data requires registration and adherence to the data use agreement.

## References

[1] Cruz-Jentoft, A. J. et al. Sarcopenia: Revised European consensus on definition and diagnosis. *Age Ageing* **48**(1), 16–31. 10.1093/ageing/afy169 (2019). 30312372 10.1093/ageing/afy169 PMC6322506

[2] Chen, L. K. et al. Asian working group for sarcopenia: 2019 Consensus update on sarcopenia diagnosis and treatment. *J. Am. Med. Dir. Assoc.* **21**(3), 300-307.e2. 10.1016/j.jamda.2019.12.012 (2020). 32033882 10.1016/j.jamda.2019.12.012

[3] Stauder, R. & Thein, S. L. Anemia in the elderly: Clinical implications and new therapeutic concepts. *Haematologica* **99**(7), 1127–1130. 10.3324/haematol.2014.109967 (2014). 24986873 10.3324/haematol.2014.109967 PMC4077071

[4] Liu, Q. et al. Hemoglobin level is negatively associated with sarcopenia and its components in Chinese aged 60 and above. *Front. Public Health* **11**, 1081843. 10.3389/fpubh.2023.1081843 (2023). 36992883 10.3389/fpubh.2023.1081843 PMC10040688

[5] Feng, Z. et al. Pathophysiological mechanisms underlying sarcopenia and sarcopenic obesity: A systematic review and meta-analysis of biomarker evidence. *Int. J. Mol. Sci.* **26**(11), 5113. 10.3390/ijms26115113 (2025). 40507924 10.3390/ijms26115113 PMC12154750

[6] Ferrucci, L. & Fabbri, E. Inflammageing: Chronic inflammation in ageing, cardiovascular disease, and frailty. *Nat. Rev. Cardiol.* **15**(9), 505–522. 10.1038/s41569-018-0064-2 (2018). 30065258 10.1038/s41569-018-0064-2 PMC6146930

[7] Lee, D. Y. & Shin, S. Sarcopenia and anemia in elderly Koreans: A nationwide population-based study. *Healthcare* **11**(17), 2428. 10.3390/healthcare11172428 (2023). 37685462 10.3390/healthcare11172428 PMC10487604

[8] Hirani, V. et al. Low hemoglobin concentrations are associated with sarcopenia, physical performance, and disability in older Australian men in cross-sectional and longitudinal analysis: The Concord Health and Ageing in Men Project. *J. Gerontol. A. Biol. Sci. Med. Sci.* **71**(12), 1667–1675. 10.1093/gerona/glw055 (2016). 26994391 10.1093/gerona/glw055

[9] Wang, H. & Lin, P. Association between sarcopenia and hemoglobin level: A systematic review and meta-analysis. *Front Med (Lausanne)* **11**, 1424227. 10.3389/fmed.2024.1424227 (2024). 39118670 10.3389/fmed.2024.1424227 PMC11306085

[10] Huang, M. et al. Serum iron level is independently associated with sarcopenia: A retrospective study. *Sci. Rep.* **14**(1), 10554. 10.1038/s41598-024-61429-0 (2024). 38719903 10.1038/s41598-024-61429-0 PMC11078979

[11] Zhao, Y., Hu, Y., Smith, J. P., Strauss, J. & Yang, G. Cohort profile: the china health and retirement longitudinal study (CHARLS). *Int. J. Epidemiol.* **43** (1), 61–68. 10.1093/ije/dys203 (2014). PMID: 23243115; PMCID: PMC3937970. 23243115 10.1093/ije/dys203 PMC3937970

[12] Steptoe, A., Breeze, E., Banks, J. & Nazroo, J. Cohort profile: The English Longitudinal Study of Ageing. *Int. J. Epidemiol.* **42**(6), 1640–1648. 10.1093/ije/dys168 (2013). 23143611 10.1093/ije/dys168 PMC3900867

[13] Camaschella, C. Iron-deficiency anemia. *N. Engl. J. Med.* **372**(19), 1832–1843. 10.1056/NEJMra1401038 (2015). 25946282 10.1056/NEJMra1401038

[14] · Pavón-Pulido, N. et al. Identification of predictors of sarcopenia in older adults using machine learning: english longitudinal study of ageing. *J. Clin. Med.* **13** (22), 6794. 10.3390/jcm13226794 (2024). PMID: 39597937; PMCID: PMC11594410. 39597937 10.3390/jcm13226794 PMC11594410

[15] Studenski, S. A. et al. The FNIH Sarcopenia Project: Rationale, study description, conference recommendations, and final estimates. *J. Gerontol. A. Biol. Sci. Med. Sci.* **69**(5), 547–558. 10.1093/gerona/glu010 (2014). 24737557 10.1093/gerona/glu010 PMC3991146

[16] Chan, K. S., Kasper, J. D., Brandt, J. & Pezzin, L. E. Measurement equivalence in ADL and IADL difficulty across international surveys of aging: Findings from the HRS, SHARE, and ELSA. *J. Gerontol. B. Psychol. Sci. Soc. Sci.* **67**(1), 121–32. 10.1093/geronb/gbr133 (2012). 22156662 10.1093/geronb/gbr133 PMC3267026

[17] · De La Cruz-Góngora, Salinas-Rodriguez, V. & Manrique-Espinoza, A. Prospective changes in anemia are associated with the incidence and persistence of sarcopenia among older mexican adults. *Front. Nutr.* **11**, 1323450. 10.3389/fnut.2024.1323450 (2024). PMID: 38544759; PMCID: PMC10967950. 38544759 10.3389/fnut.2024.1323450 PMC10967950

[18] · Yoo, J. J. et al. Biomarkers of erythropoiesis response to intravenous iron in a crossover pilot study in unexplained anemia of the elderly. *Hematology* **28** (1), 1–8. 10.1080/16078454.2023.2204613 (2023). PMID: 37114660; PMCID: PMC10281558. 37114660 10.1080/16078454.2023.2204613 PMC10281558

[19] · Kim, Y. H. et al. The estimated mediating roles of anemia-related variables in the association between kidney function and mortality: a national health and nutrition examination survey (NHANES) study. *Sci. Rep.* **14** (1), 6621. 10.1038/s41598-024-56877-7 (2024). PMID: 38503784; PMCID: PMC10951385. 38503784 10.1038/s41598-024-56877-7 PMC10951385

[20] · Wacka, E., Nicikowski, J., Jarmuzek, P. & Zembron-Lacny, A. Anemia and its connections to inflammation in older adults: a review. *J. Clin. Med.* **13** (7), 2049. 10.3390/jcm13072049 (2024). PMID: 38610814; PMCID: PMC11012269. 38610814 10.3390/jcm13072049 PMC11012269

[21] · Wang, P. et al. Association between dietary patterns and anemia in older adults: the 2015 china adults chronic diseases and nutrition surveillance. *BMC Public. Health*. **25** (1), 1072. 10.1186/s12889-025-22199-0 (2025). PMID: 40108541; PMCID: PMC11924724. 40108541 10.1186/s12889-025-22199-0 PMC11924724

[22] Lukito, W. & Wahlqvist, M. L. Intersectoral and eco-nutritional approaches to resolve persistent anemia in Indonesia. *Asia Pac. J. Clin. Nutr.* **29**(Suppl 1), S1–S8. 10.6133/apjcn.202012_29(S1).01 (2020). 33377742 10.6133/apjcn.202012_29(S1).01

[23] · Lipoeto, N. I. & Masrul, Nindrea, R. D. Nutritional contributors to maternal anemia in indonesia: chronic energy deficiency and micronutrients. *Asia Pac J Clin Nutr.* **29**(Suppl 1): S9-S17. (2020). 10.6133/apjcn.202012_29(S1).02. PMID: 33377743. 10.6133/apjcn.202012_29(S1).02 33377743

[24] Pathania, A. et al. Prevalence of anemia among elderly persons residing in old age homes in national capital territory, Delhi, India. *Indian J. Public Health.* **63**(4), 288–292. 10.4103/ijph.IJPH_412_18 (2019). 32189646 10.4103/ijph.IJPH_412_18

[25] Hwang, Y. et al. Anemia prevalence time trends and disparities in the US population: Examination of NHANES 1999–2020. *J. Investig. Med.* **71**(3), 286–294. 10.1177/10815589221140597 (2023). 36803039 10.1177/10815589221140597

[26] · Wang, P. et al. Analysis of prevalence, years lived with disability, and trends of anemia burden and main causes in china. *Front. Public. Health*. **13**, 1564756. 10.3389/fpubh.2025.1564756 (2025). PMID: 40510576; PMCID: PMC12158651. 40510576 10.3389/fpubh.2025.1564756 PMC12158651

[27] de Smalen, L. M. et al. Impaired age-associated mitochondrial translation is mitigated by exercise and PGC-1α. *Proc. Natl. Acad. Sci. U. S. A.* **120**(36), e2302360120. 10.1073/pnas.2302360120 (2023). 37639610 10.1073/pnas.2302360120 PMC10483666

[28] Geraci, A. et al. Sarcopenia and menopause: The role of estradiol. *Front Endocrinol (Lausanne)* **12**, 682012. 10.3389/fendo.2021.682012 (2021). 34093446 10.3389/fendo.2021.682012 PMC8170301

[29] Meng, Y. et al. Relationship of anabolic and catabolic biomarkers with muscle strength and physical performance in older adults: A population-based cross-sectional study. *BMC. Musculoskelet Disord.* **16**, 202. 10.1186/s12891-015-0654-7 (2015). 26286594 10.1186/s12891-015-0654-7 PMC4545782

[30] Granic, A., Sayer, A. A., Cooper, R. & Robinson, S. M. Nutrition in the prevention and treatment of skeletal muscle ageing and sarcopenia: A single nutrient, a whole food and a whole diet approach. *Proc. Nutr. Soc.* **84**(4), 340–355. 10.1017/S0029665124007432 (2025). 39417264 10.1017/S0029665124007432 PMC7616828

[31] Lee, E., Kim, I. D. & Lim, S. T. Physical activity and protein-intake strategies to prevent sarcopenia in older people. *Int. Health.* **17**(4), 423–430. 10.1093/inthealth/ihae064 (2025). 40248876 10.1093/inthealth/ihae064 PMC12212230

[32] Feng, H., Wang, H. & Zhou, W. Comment on “bidirectional transitions of sarcopenia states in older adults: the longitudinal evidence from CHARLS” by Luo et al.. *J. Cachexia Sarcopenia Muscle.* **15**(6), 2875–2876. 10.1002/jcsm.13593 (2024). 39344683 10.1002/jcsm.13593 PMC11634473

[33] Szlejf, C., Suemoto, C. K., Lotufo, P. A. & Benseñor, I. M. Association of sarcopenia with performance on multiple cognitive domains: Results from the ELSA-Brasil study. *J. Gerontol. A Biol. Sci. Med. Sci.* **74**(11), 1805–1811. 10.1093/gerona/glz118 (2019). 31058914 10.1093/gerona/glz118

