# Supplementary material for: Anemia as an independent risk factor for sarcopenia in older adults: a cohort study based on CHARLS and ELSA
Source: Sci Rep. 2026 May 27;16:24127. doi: 10.1038/s41598-026-54626-6 (PMC13438801; doi:10.1038/s41598-026-54626-6)
Supplement: Supplementary file 1 [file 41598_2026_54626_MOESM1_ESM.pdf]

Table S1. Subgroup analysis of the association between anemia at baseline and the risk of incident sarcopenia in the CHARLS cohort.

Stratification	Subgroup	N	HR (95% CI)	p-value
**Sex**	Male	725	2.08 (1.06-4.05)	0.032
	Female	682	1.32 (0.68-2.58)	0.411
**Drinking**	Ever drinker	403	2.18 (0.84-5.67)	0.109
	Never drinker	935	1.64 (0.95-2.86)	0.078
**Smoking**	Ever smoker	571	1.83 (0.85-3.92)	0.121
	Never smoker	829	1.58 (0.87-2.87)	0.132
**Obesity**	BMI ≥24	614	2.07 (0.89-4.85)	0.093
	BMI <24	717	1.30 (0.74-2.29)	0.354

Abbreviations: BMI, body mass index; CHARLS, China Health and Retirement Longitudinal Study; CI, confidence interval; HR, hazard ratio.

Note: Hazard ratios and 95% CIs were derived from Cox proportional hazards models adjusted for sex, annual household economic status, marital status, education status, number of chronic conditions, body mass index, smoking status, drinking status, and ADL disability score (where applicable). Obesity was defined as BMI ≥ 24 kg/m² according to Asian criteria. P-values were two-sided, and $P < 0.05$ was considered statistically significant.
