# Supplementary material for: Anemia as an independent risk factor for sarcopenia in older adults: a cohort study based on CHARLS and ELSA
Source: Sci Rep. 2026 May 27;16:24127. doi: 10.1038/s41598-026-54626-6 (PMC13438801; doi:10.1038/s41598-026-54626-6)
Supplement: Supplementary file 2 [file 41598_2026_54626_MOESM2_ESM.pdf]

Table S2. Subgroup analysis of the association between anemia at baseline and the risk of incident sarcopenia in the ELSA cohort.

Stratification	Subgroup	N	HR (95% CI)	p-value
**Sex**	Male	1331	0.98 (0.24-4.02)	0.979
	Female	1590	4.72 (2.25-9.93)	<0.001
**Drinking**	Ever drinker	2529	2.53 (1.17-5.46)	0.019
	Never drinker	189	3.83 (0.86-17.00)	0.077
**Smoking**	Ever smoker	1680	2.77 (1.20-6.40)	0.017
	Never smoker	1238	2.61 (0.94-7.28)	0.067
**Obesity**	BMI ≥25	2101	3.03 (1.58-5.79)	0.001
	BMI <25	820	—	0.999

Abbreviations: BMI, body mass index; CI, confidence interval; ELSA, English Longitudinal Study of Ageing; HR, hazard ratio.

Note: Hazard ratios and 95% CIs were derived from Cox proportional hazards models adjusted for sex, marital status, education status, annual household economic status, number of chronic conditions, smoking status, drinking status, and ADL disability score. Obesity was defined as BMI ≥ 25 kg/m² according to WHO criteria. The dash (—) indicates that valid HR could not be estimated due to no events in the anemic subgroup. P-values were two-sided, and $P < 0.05$ was considered statistically significant.
