# Supplementary material for: Anemia as an independent risk factor for sarcopenia in older adults: a cohort study based on CHARLS and ELSA
Source: Sci Rep. 2026 May 27;16:24127. doi: 10.1038/s41598-026-54626-6 (PMC13438801; doi:10.1038/s41598-026-54626-6)
Supplement: Supplementary file 3 [file 41598_2026_54626_MOESM3_ESM.pdf]

Table S3. Sensitivity analysis of the association between baseline anemia and incident sarcopenia in the CHARLS cohort

Model	Hazard Ratio (HR)	95% Confidence Interval	p-value
Model 1	1.51	1.16 – 1.97	0.002
Model 2	1.61	1.23 – 2.11	<0.001
Model 3	1.53	1.17 – 2.01	0.002

Note: Model 1: Unadjusted. Model 2: Adjusted for sex, marital status, education status, and annual household economic status. Model 3: Additionally adjusted for number of chronic conditions, body mass index (BMI), smoking status, drinking status, and activities of daily living (ADL) disability score. P-values were two-sided, and $P < 0.05$ was considered statistically significant.
