# Supplementary material for: Anemia as an independent risk factor for sarcopenia in older adults: a cohort study based on CHARLS and ELSA
Source: Sci Rep. 2026 May 27;16:24127. doi: 10.1038/s41598-026-54626-6 (PMC13438801; doi:10.1038/s41598-026-54626-6)
Supplement: Supplementary file 4 [file 41598_2026_54626_MOESM4_ESM.pdf]

Table S4. Sensitivity analysis of the association between baseline anemia and incident sarcopenia in the ELSA cohort

Model	Hazard Ratio (HR)	95% Confidence Interval	p-value
Model 1	2.70	1.41 – 5.16	0.003
Model 2	2.77	1.39 – 5.52	0.004
Model 3	2.59	1.30 – 5.18	0.007

Note: Note: Model 1: Unadjusted. Model 2: Adjusted for sex, marital status, education status, and annual household economic status. Model 3: Additionally adjusted for number of chronic conditions, body mass index (BMI), smoking status, drinking status, and activities of daily living (ADL) disability score. P-values were two-sided, and $P < 0.05$ was considered statistically significant.
