# Supplementary material for: Anemia as an independent risk factor for sarcopenia in older adults: a cohort study based on CHARLS and ELSA
Source: Sci Rep. 2026 May 27;16:24127. doi: 10.1038/s41598-026-54626-6 (PMC13438801; doi:10.1038/s41598-026-54626-6)
Supplement: Supplementary file 5 [file 41598_2026_54626_MOESM5_ESM.pdf]

Figure S5. Forest plot of hazard ratios for the association between baseline anemia and incident sarcopenia in the CHARLS cohort

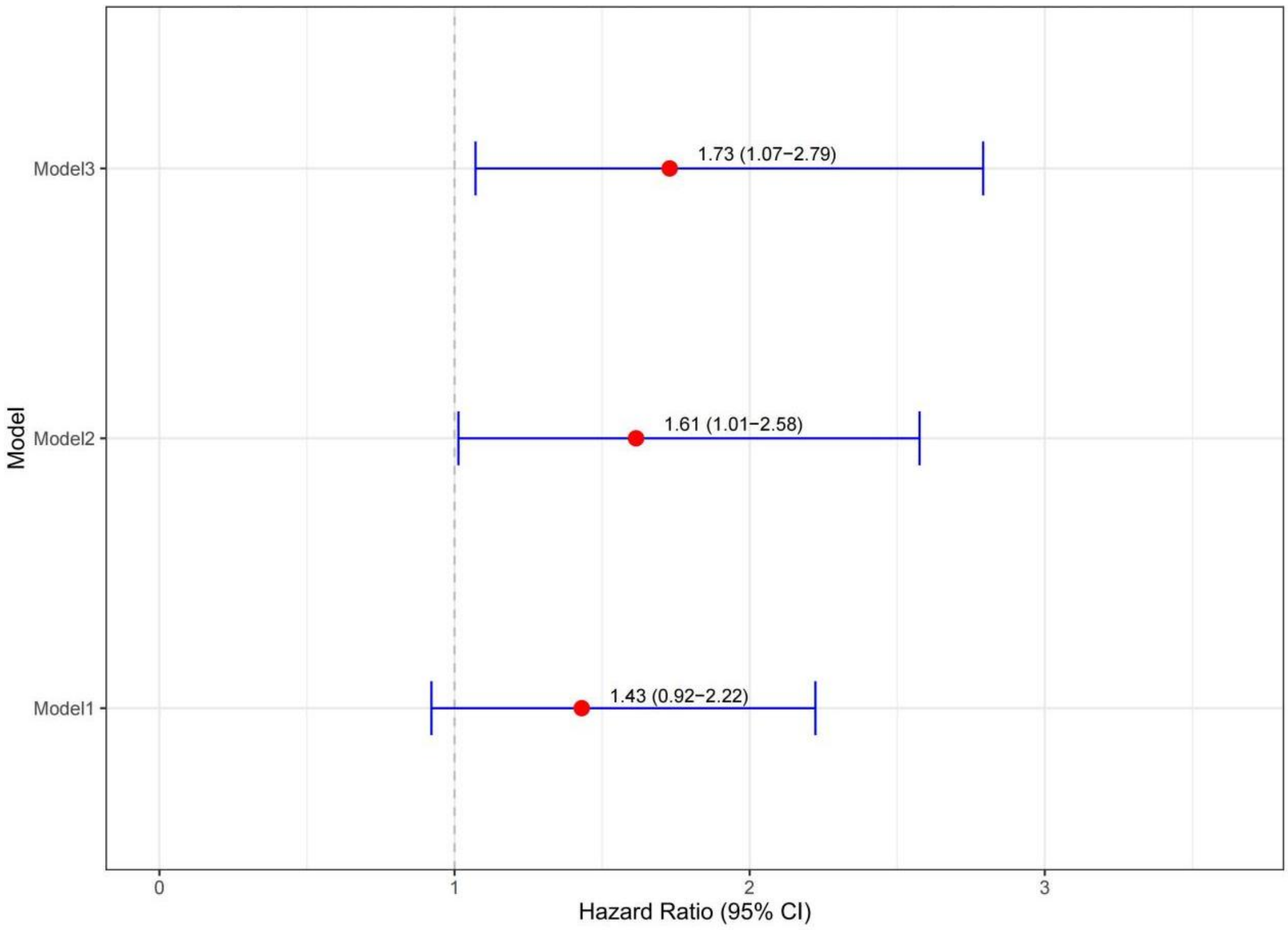

Hazard ratios (HRs) and 95% confidence intervals (CIs) for the association between baseline anemia and incident sarcopenia in the CHARLS cohort.

Model 1: Unadjusted.

Model 2: Adjusted for sex, marital status, education status, and annual household economic status.

Model 3:Additionally adjusted for number of chronic conditions, body mass index (BMI), smoking status, drinking status, and activities of daily living (ADL) disability score.
