# Supplementary material for: Anemia as an independent risk factor for sarcopenia in older adults: a cohort study based on CHARLS and ELSA
Source: Sci Rep. 2026 May 27;16:24127. doi: 10.1038/s41598-026-54626-6 (PMC13438801; doi:10.1038/s41598-026-54626-6)
Supplement: Supplementary file 7 [file 41598_2026_54626_MOESM7_ESM.pdf]

Figure S7.Subgroup analyses of the association between baseline anemia and incident sarcopenia in the CHARLS cohort (full adjustment model)

Subgroup		N	HR (95%cl)	p for interaction
Sex	Male	725	2.08 (1.06−4.05)	0.156
	Female	682	1.32 (0.68−2.58)	
Drinking	Drinker	403	2.18 (0.84−5.67)	0.294
	Non−drinker	935	1.64 (0.95−2.86)	
Smoking	Smoker	571	1.83 (0.85−3.92)	0.687
	Non−smoker	829	1.58 (0.87−2.87)	
Obesity	BMI>=24	614	2.07 (0.89−4.85)	0.599
	BMI<24	717	1.30 (0.74−2.29)	

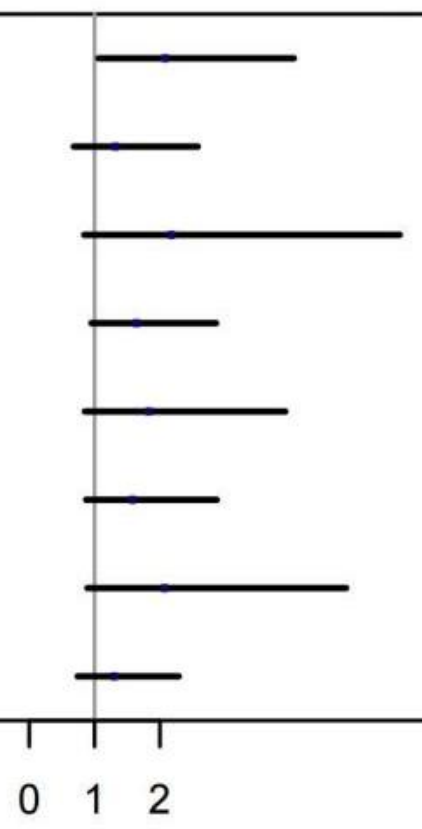

Hazard ratios (HRs) and 95% confidence intervals (CIs) for the association between baseline anemia and incident sarcopenia, stratified by sex, drinking status, smoking status, and obesity (BMI ≥ 24 kg/m² vs. BMI < 24 kg/m²) in the CHARLS cohort.

Analyses were performed under the full adjustment model, adjusted for sex, annual household economic status, marital status, education status, number of chronic conditions, body mass index, smoking status, drinking status, and ADL disability score.

P for interaction was calculated to examine the potential effect modification between anemia and each stratifying variable.
