# Supplementary material for: Anemia as an independent risk factor for sarcopenia in older adults: a cohort study based on CHARLS and ELSA
Source: Sci Rep. 2026 May 27;16:24127. doi: 10.1038/s41598-026-54626-6 (PMC13438801; doi:10.1038/s41598-026-54626-6)
Supplement: Supplementary file 8 [file 41598_2026_54626_MOESM8_ESM.pdf]

Figure S8.Subgroup analyses of the association between baseline anemia and incident sarcopenia in the ELSA cohort (full adjustment model)

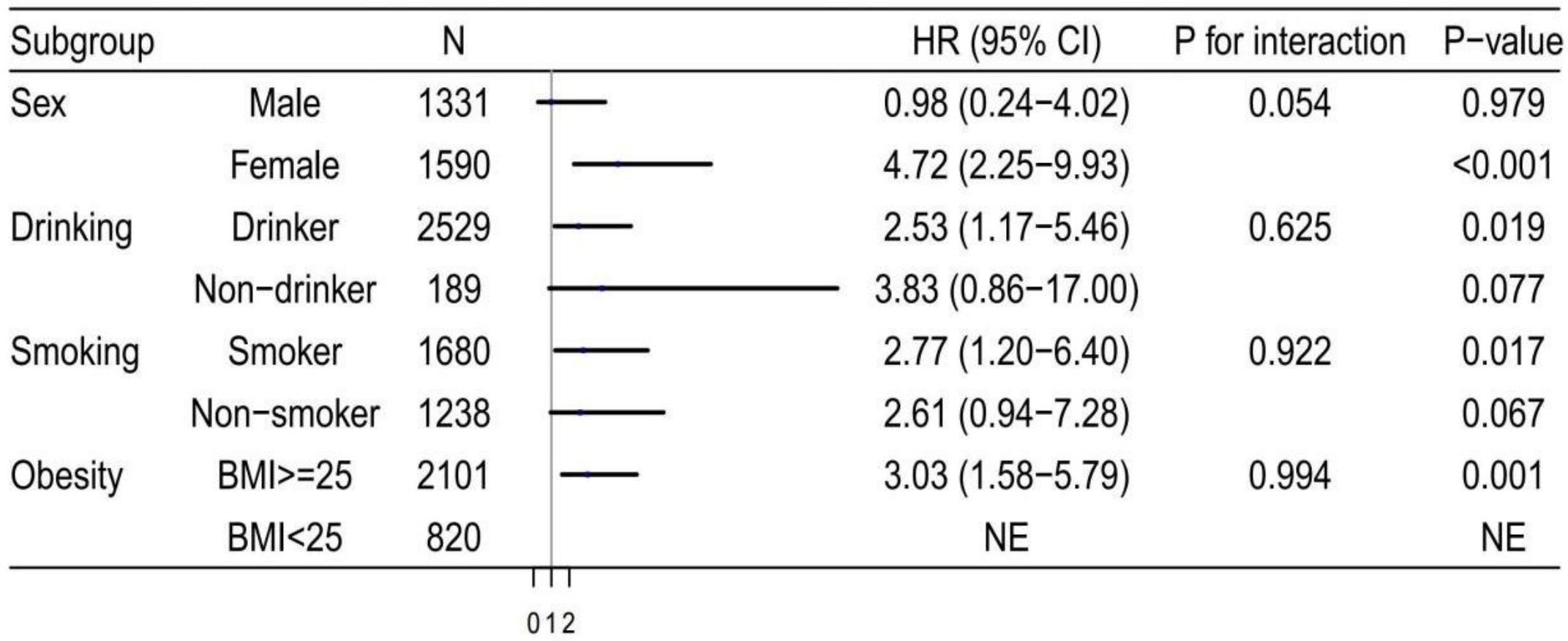


Hazard ratios (HRs) and 95% confidence intervals (CIs) for the association between baseline anemia and incident sarcopenia, stratified by sex, drinking status, smoking status, and obesity (BMI ≥ 25 kg/m² vs. BMI < 25 kg/m²) in the ELSA cohort.

Analyses were performed under the full adjustment model, adjusted for sex, marital status, education status, annual household economic status, number of chronic conditions, smoking status, drinking status, and ADL disability score.

NE, not estimable. For the BMI < 25 kg/m² subgroup, the effect estimate was not estimable due to the small number of anemic participants and incident sarcopenia events within this stratum, which led to insufficient statistical precision for model convergence.

P for interaction was calculated to examine the potential effect modification between anemia and each stratifying variable.
